# Personalized Approach to Severe Asthma

**DOI:** 10.1155/2018/2465172

**Published:** 2018-12-24

**Authors:** Enrico Heffler, Giorgio Walter Canonica, Zuzana Diamant, Joao Fonseca, Andrei Malinovschi

**Affiliations:** ^1^Personalized Medicine, Asthma and Allergy, Humanitas Clinical and Research Center, IRCCS, Rozzano (MI), Italy; ^2^Department of Biomedical Sciences, Humanitas University, Pieve Emanuele (MI), Italy; ^3^Institute for Clinical Science, Skane University Hospital, Deptartment of Respiratory Medicine & Allergology, Lund, Sweden; ^4^Department of Clinical Pharmacy & Pharmacology and Deptartment of General Practice, UMCG, and QPS-NL, Groningen, Netherlands; ^5^Department of Community Medicine, Information and Decision Sciences & CINTESIS, Faculdade de Medicina da Universidade do Porto and MEDIDA, Lda, Porto, Portugal; ^6^Department of Medical Sciences, Clinical Physiology, Uppsala University, Uppsala, Sweden

Approximately 5-10% of asthmatics are barely controlled or clinically and/or functionally uncontrolled despite a high dose of inhaled corticosteroids (ICS) plus another controller agent (e.g., long-acting beta2-agonists, LABA; leukotriene-receptor antagonists, LTRA; long-acting muscarinic agents, LAMA) or maintenance oral corticosteroid therapy. These patients are defined as affected by “severe asthma” according to the most recent recommendations of the European Respiratory Society (ERS) and the American Thoracic Society [1]. The diagnosis of severe asthma is made after having ruled out or having treated clinical conditions that may mimic asthmatic symptoms (e.g., extra-thoracic hyperresponsiveness syndromes, vocal cord dysfunction), comorbidities that may worsen disease control (e.g., allergic or nonallergic rhinitis, chronic rhinosinusitis with or without nasal polyps, bronchiectasis, and gastroesophageal reflux), possible incorrect inhaler techniques, and/or poor treatment adherence. During the past decade, advanced research brought insight into the heterogeneous mechanisms of severe asthma and helped to reveal several potential therapeutic targets [2].

Following the introduction of the first available biologic agents in clinical practice, the way of diagnosing and managing the majority of patients with severe asthma dramatically changed from a “one-size-fits-all” approach to precision medicine [3]. Presently, we are experiencing a new era in the management of severe asthmatic patients, as subjects are clinically characterized in phenotypes [4] or in treatable traits [5] in order to personalize their disease-management.

In this special issue, the latest knowledge and novel findings in severe asthma pathogenesis, pheno/endotyping and management with a particular focus on personalized and precision medicine approaches, have been addressed.

The classification of patients according to their phenotypes and/or endotypes [4] is strictly dependent on the identification of reliable biomarkers, ideally noninvasive and available for point-of-care [6]. The article by E. Mortaz et al. elegantly summarizes a plethora of possible new biomarkers from tissue-derived exosomes. These small membrane-enclosed vesicles contain mRNA and miRNA, lipids, and a vast array of different proteins depending on their cell of origin. Furthermore, exosomes may also be potentially used for developing novel therapeutic strategies. C. Galeone et al. pointed their attention on how the new field of “omics” sciences (including proteomics, metabolomics, transcriptomics, and genomics) may provide new biomarkers, novel targets for diagnostic tests, and pharmacological treatments.

This complex scenario of new technologies and biomarkers, applied to the process of identification of specific severe asthma phenotypes and endotypes, is part of the precision medicine approach to asthma. The direct consequence of a better characterization of patients under the immunological point of view is the possibility to treat them with novel biologic agents, acting directly towards those immunological mechanisms that are involved in every single endotype of severe asthma [7]. The first available biologic agent for severe asthma was omalizumab, a fully humanized anti-IgE monoclonal antibody. Its clinical efficacy and effectiveness in severe allergic patients have been proved extensively. There are some recent studies also suggesting effectiveness in nonallergic severe asthmatics. C. C. Loureiro et al. reviewed the current evidence on both of these possible uses of omalizumab in this present Special Issue. In the past few years, novel therapeutic targets have been addressed by recently approved biologic agents: mainly, anti-IL5 strategies are currently worldwide used for severe eosinophilic asthma [8]. D. Bagnasco et al. overviewed the possible molecular targets and related biologic drugs, blocking the IL5-mediated eosinophilic inflammation in severe asthma. C. Pelaia et al. dedicated their review article to the specific mechanism and clinical effects of benralizumab, an afucosylated monoclonal antibody towards the IL5-receptor, a newly approved drug for treatment of severe asthma in 2018. This drug has a remarkable affinity for the Fc*γ*RIIIa receptor of NK cells that gives to the drug the ability to induce the apoptotic mechanism named antibody-dependent cell-mediated cytotoxicity (ADCC). L. Brussino et al. critically revised the published literature on anti-IL5 treatment in asthma and highlighted the still present unmet needs, critical points, and open questions on efficacy and real-life effectiveness of this category of drugs in severe asthmatics.

Beyond the use of biologic agents, among the approved drugs for severe asthma, the inhaled LAMA tiotropium may play a role, both before starting any biologic treatment and for those patients not meeting the indication for any of the currently available biologics. E. Hamelmann et al. reviewed the evidence of the use of inhaled tiotropium in severe asthmatics.

A critical point for all treatments for severe asthma is the* a priori* identification of responder patients: this depends on many variables (e.g., clinical, functional and immunological characteristics, associated comorbidities) that may not correspond precisely to the features of the extremely selected patients included into randomized-controlled trials. Therefore, real-life big-data on severe asthma are needed to improve the characterization of our patients and provide them with adequate and tailored treatment. A very effective approach to obtain real-life big-data is establishing national and international registries [9, 10], as they will include a large amount of information on “real” patients managed by physicians in their daily clinical activity. A. Sá-Sousa et al., in this special issue, described the protocol and the aims of such an initiative, the Portuguese Severe Asthma Registry.

In conclusion, this special issue updates and summarizes recent knowledge on many different aspects of severe asthma.
